# Emergency Medical Team Response during the Hokkaido Eastern Iburi Earthquake 2018: J-SPEED Data Analysis

**DOI:** 10.1017/S1049023X23000432

**Published:** 2023-06

**Authors:** Yui Yumiya, Odgerel Chimed-Ochir, Ryoma Kayano, Yoshiaki Hitomi, Kouki Akahoshi, Hisayoshi Kondo, Akinori Wakai, Seiji Mimura, Kayako Chishima, Yoshiki Toyokuni, Yuichi Koido, Tatsuhiko Kubo

**Affiliations:** 1. Department of Public Health and Health Policy, Graduate School of Biomedical and Health Sciences, Hiroshima University, Hiroshima, Japan; 2. World Health Organization Centre for Health Development (WHO Kobe Centre), Kobe, Japan; 3. Hokkaido Government Department of Health and Welfare, Hokkaido, Japan; 4. National Hospital Organization Headquarters DMAT Secretariat MHLW Japan, Tokyo, Japan

**Keywords:** Emergency Medical Team, Emergency Medical Team minimum data set, Hokkaido Eastern Iburi Earthquake, Japan, J-SPEED

## Abstract

**Introduction:**

In the last ten years, Japan has experienced several large-scale earthquakes with devastating social and health impacts. Earthquakes directly and indirectly cause a variety of health problems. Further investigation is required to increase preparedness and preventive efforts. In response to the Hokkaido Eastern Iburi Earthquake on September 6, 2018, 32 Emergency Medical Teams (EMTs) employed the Japanese version of Surveillance in Post-Extreme Emergencies and Disasters (J-SPEED) as a national standard daily reporting template, gathering data on the number and type of health problems treated.

**Study Objective:**

The purpose of the study is to conduct a descriptive epidemiology study using the J-SPEED data to better understand the health problems during the earthquake disaster.

**Methods:**

Reported items in J-SPEED (Ver 1.0) form were analyzed by age, gender, and time to better understand the health issues that have arisen from the earthquake.

**Results:**

Most consultations (721; 97.6%) occurred between Day 1 and Day 13 of the 32-day EMT response. During the response period, disaster stress-related symptoms were the most common health event (15.2%), followed by wounds (14.5%) and skin diseases (7.0%).

**Conclusion:**

The most often reported health event during the response period was stress-associated illnesses related to disasters, followed by wounds and skin conditions. The health consequences of natural disasters depend on diverse local environment and population. As a result, this initial study was hard to generalize; however, it is expected that data accumulated using the J-SPEED system in the future will strengthen and extend the conclusions.

## Introduction

Disaster events have increased over the past several decades. Earthquakes account for only approximately eight percent of all disasters and disaster-related deaths world-wide,^1^ whereas they were responsible for 18% over the past three decades, 1985 to 2018, in Japan.^2^ In the last ten years, Japan experienced three large-scale earthquakes: the Great East Japan Earthquake in 2011 (magnitude 9.0), the Kumamoto Earthquake in 2016 (magnitude 7.3), and the Hokkaido Eastern Iburi Earthquake in 2018 (magnitude 6.7).^3^ The devastating social and health impacts left in the wake of these disasters make prevention and preparedness a crucial challenge in Japan.

Earthquakes directly and indirectly inflict a wide range of health problems, including trauma, infectious diseases, exacerbation of pre-existing medical conditions, and psychological problems.^4^ A detailed accounting of health issues and specific events associated with earthquakes will promote preparedness and preventive efforts.^5^ However, due to various inhibiting factors such as environmental risks, logistical limits, political and economic challenges, communication difficulties among stakeholders, and cultural barriers, gathering health data during disasters is a huge task.^6^

Emergency Medical Teams (EMTs) treating emergency and disaster victims are made up of doctors, nurses, paramedics, support workers, and logisticians. They give direct clinical care to patients and are a critical part of health care during a disaster. The demand for standardized EMT daily reporting emerged as a result of the Great East Japan Earthquake in 2011. The newly developed Joint Committee for Disaster Medical Recording (Japan) proposed the standard disaster medical record and daily reporting forms called the Japan - Surveillance in Post-Extreme Emergencies and Disasters (J-SPEED).^7^ The J-SPEED is based on the Surveillance in Post Extreme Emergencies and Disasters (SPEED) system, developed by the Department of Health of the Republic of the Philippines, to be used as a standard method of medical reporting by front-line health care workers to collect near real-time health data during emergencies and disasters.^6,8^

The Hokkaido Eastern Iburi Earthquake occurred on September 6, 2018, with landslides and a blackout across Hokkaido as secondary disasters. Human casualties included 44 dead, 51 seriously injured, and 734 with minor/medium injuries. Four hundred sixty-two (462) houses were totally destroyed, 1,570 were partially destroyed, and an additional 12,600 were damaged.^9^ The EMTs were dispatched to the affected areas immediately after the disaster on September 6, 2018 and began medical relief activities. The J-SPEED was activated as the daily reporting mechanism, gathering and communicating information on the number and type of patients treated with the EMT Coordination Cell (EMTCC). The aim of the epidemiological study is to describe the detailed and timely J-SPEED data to better understand the health issues that arise during the course of earthquake disasters.

## Methods

### Data Collection

From September 6 - October 7, 2018, data were gathered on the quantity and types of health conditions handled by EMTs in accordance with J-SPEED (Version 1.0). On a daily basis, each EMT delivered a report of patient consultations to the EMTCC, where the data were collated. The J-SPEED (Ver1.0) form defines 26 items to be recorded, such as demographic characteristics and primary and relevant secondary health events related to EMT operations. Moreover, EMTCC may define up to four additional items during a disaster response, customized to the disaster’s specific circumstances. During the earthquake, EMTs reported bruises, constipation, and headache, which EMTCC indicated as additional symptoms.

During the earthquake on September 6, 2018 in Hokkaido, a total of 741 consultations were recorded by 32 EMTs. Among them, only two deaths were reported. Therefore, the deaths from analysis were excluded, thus 739 consultations remained for analysis.

### Data Analysis

Age, gender, and time were used to examine all items reported on the J-SPEED form. The J-SPEED was offered in two different formats: paper and a mobile application. Of all consultations, approximately 97% of the J-SPEED mobile applications were used during this Hokkaido Iburi Earthquake. The age groups on the mobile application are: 0, 1-14, 15-64, and ≥65; however, the age groups in the paper form are: 0, 1-8, 9-74, and ≥75. Age groups of 0, 1-8, 9-74, and ≥75 were converted to 0, 1-14, 15-64, and ≥65 to combine the age categories of the two J-SPEED formats. Furthermore, because the number of health issues attributed to zero-year-old patients represented only 0.8% (N = 6) of overall health events, the age categories 0 and 1-15 were combined. All analyses were conducted using Microsoft Excel (Microsoft Corp.; Redmond, Washington USA) and STATA v15.1 (STATA Corp; College Station, Texas USA).

### Ethical Consideration

Hiroshima University (Hiroshima, Japan) Ethics Committee approved this study (No. E-2059). This study was funded by the World Health Organization (WHO; Geneva, Switzerland) Kobe Centre for Health Development (WKC-HEDRM-K19009), Grants for Research on Policy Planning and Evaluation from the Ministry of Health, Labor, and Welfare, Japan (grant numbers:19IA2014, 20CA2063), and JSPS KAKENHI Grant Number JP21K09020.

## Results

**Figure 1.**
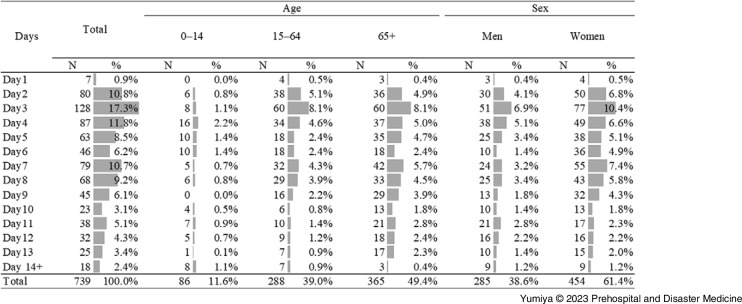
Demographic Information for Consultations by Emergency Medical Teams during the Hokkaido Eastern Iburi Earthquake, 2018. Note: The number of daily consultations by the age and gender distribution are shown.

The number of daily consultations by the age and gender distribution are shown in Figure 1. A total of 739 consultations, made of 26 types, were recorded over the 32-day EMT response. The majority of consultations (721; 97.6%) occurred between Day 1 and Day 13, reaching a peak on Day 3 with 128 consultations (17.3% of total consultations), then declining until Day 13, after which reporting remained steady through Day 32. People over the age of 65 (49.4%) received medical consultations more than those aged 0-14 (11.6%) and 15-64 (39.0%).

Regarding gender, women accounted for 61.4% and men 38.6% of consultations. Table 1 shows the types of health events reported by EMTs. Out of the 739 consultations recorded, and excluding other health events, disaster stress-related symptoms accounted for the highest percentage (112/15.2%), followed by wounds (107/14.5%), skin disease (52/7.0%), hypertension (41/5.5%), acute respiratory infections (ARIs; 26/3.5%), and constipation (26/3.5%). Examining types of consultations across age groups, the highest prevalence in children, ages 0-14 years, was skin diseases, ARI, bruises, and non-disaster-related events; disaster stress-related symptoms, wounds, headache, urgent need for psychological support, burn, dialysis, bronchial asthma, and need for transfer were most common among adults, ages 15-64 years; blood pressure, constipation, fever, gastrointestinal infections, deep vein thrombosis (DVT), other, triage yellow or over, and interrupted essential medications were prevalent in people over 65 years old.

**Table 1.** Health Events Reported by Emergency Medical Teams during the Hokkaido Eastern Iburi Earthquake, 2018

Health Events/Age Group (years old)	Total		0-14		15-64		65+	
	N	%	N	%^a^	N	%	N	%
Total Number of Consultations	739	(100%)	86	(11.6%)	288	(39.0%)	365	(49.4%)
**Health Events**								
Disaster Stress-Related Symptoms	112	[15.2%]	10	[11.6%]	62	[21.5%]	40	[11.0%]
Wound	107	[14.5%]	8	[9.3%]	48	[16.7%]	51	[14.0%]
Skin Diseases	52	[7.0%]	13	[15.1%]	22	[7.6%]	17	[4.7%]
Hypertension	41	[5.5%]	0	[0.0%]	10	[3.5%]	31	[8.5%]
Acute Respiratory Infections	26	[3.5%]	6	[7.0%]	13	[4.5%]	7	[1.9%]
Constipation	26	[3.5%]	3	[3.5%]	4	[1.4%]	19	[5.2%]
Bruise	21	[2.8%]	5	[5.8%]	9	[3.1%]	7	[1.9%]
Fever	14	[1.9%]	2	[2.3%]	3	[1.0%]	9	[2.5%]
Gastrointestinal Infections	13	[1.8%]	2	[2.3%]	2	[0.7%]	9	[2.5%]
Headache	10	[1.4%]	0	[0.0%]	7	[2.4%]	3	[0.8%]
Deep Vein Thrombosis	9	[1.2%]	0	[0.0%]	3	[1.0%]	6	[1.6%]
Urgent Need for Psychological Support	8	[1.1%]	0	[0.0%]	4	[1.4%]	4	[1.1%]
Fracture	5	[0.7%]	0	[0.0%]	1	[0.3%]	4	[1.1%]
Burn	5	[0.7%]	0	[0.0%]	5	[1.7%]	0	[0.0%]
Dialysis	4	[0.5%]	0	[0.0%]	2	[0.7%]	2	[0.5%]
Bronchial Asthma	3	[0.4%]	0	[0.0%]	2	[0.7%]	1	[0.3%]
Other	12	[1.6%]	1	[1.2%]	4	[1.4%]	7	[1.9%]
**Other Events**								
Triage	27	[3.7%]	3	[3.5%]	9	[3.1%]	15	[4.1%]
Need for Transfer	9	[1.2%]	0	[0.0%]	5	[1.7%]	4	[1.1%]
Interrupted Essential Medications	7	[0.9%]	0	[0.0%]	2	[0.7%]	5	[1.4%]
Non-Disaster-Related Events	118	[16.0%]	15	[17.4%]	41	[14.2%]	62	[17.0%]

Note: The events with less than three cases were not shown in the table: emergency care needs (1), emergency drinking water and food supply needs (0), urgent nutritional support needs (0), suspected measles (0), suspected tetanus (0), drowning (1), and crash syndrome (1). Events are listed in accordance with the order of number of cases.

a (%) - Percentage of age group within specific event; [%] - Percentage of specific event within age group.

**Figure 2.**
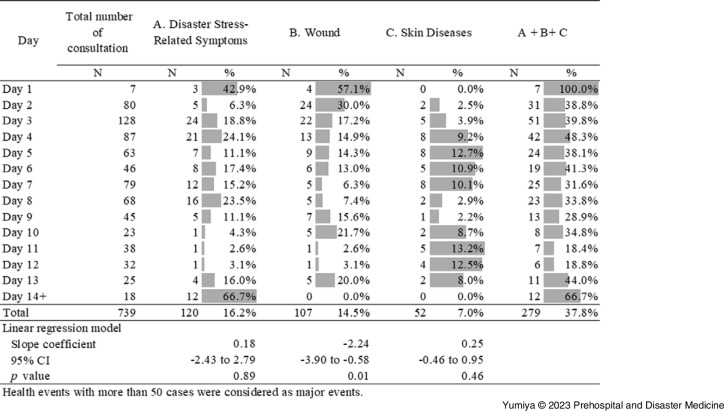
Three Major Health Events by Period Reported by Emergency Medical Teams during the Hokkaido Eastern Iburi Earthquake, 2018. Note: The three major health events, disaster stress-related symptoms, wounds, and skin diseases, are described according to period of Emergency Medical Team operation.

The three major health events, disaster stress-related symptoms, wounds, and skin diseases, are described according to period of EMTs operation in Figure 2. The absolute number shows the number of consultations for each day and the percent shows the proportion of diseases among corresponding numbers of consultations for each day. The three major health events in two weeks account for more than one-third of all health events, except Day 9, Day 11, and Day 12. The majority of consultations of three health events were provided on Day 3 in the first week after EMTs arrived. The percentage of wounds out of total consultations statistically decreased, whereas stress and skin diseases did not statistically decrease over time.

## Discussion

According to the analysis of J-SPEED data for Hokkaido Eastern Iburi Earthquake in 2018, 32 EMTs delivered a total of 739 consultations, with the maximum number of 721 (97.6%) consultations provided between Day 1 and Day 13 after EMTs arrived. The percentage of health consultations was higher for women (61.4%) than for men (38.6%), and was highest with elderly people (49.4%), followed by adults (39.0%) and children (11.6%) by age group. The most common health event was disaster stress-related symptoms (15.2%), followed by wounds (14.5%) and skin diseases (7.0%).

Data and information for emergency medical responses and natural disaster literature have traditionally been collected from a variety of sources, such as medical records from secondary and tertiary hospitals, victims’ interviews, death certificates, emergency medical data, and other retrospective data.^10,11^ The J-SPEED data used in this study were collected immediately via EMTs dispatched on-site, and visualized as a daily report, making this a one-of-a-kind study when the earthquake occurred.

The health effects of earthquakes are dependent on various factors, including the type of earthquake, its magnitude, geography, the earthquake’s secondary effects (such as landslides, tsunamis, and fires), patterns of exposure, weather conditions, hygiene, the population’s underlying vulnerability, culture, and country’s emergency preparedness. Therefore, the health impacts of a particular earthquake event are context-specific and are very different between developed and developing countries, for example. The observations with as much of the available evidence were attempted to present, aiming to understand how Japan-specific characteristics of earthquake-related health problems may be avoided, and developed best practices for Japan and other countries.

Examining individual attributes across all recorded events, the findings are consistent with previous studies in that the percentage of health consultations is higher for vulnerable groups such as women and the elderly (65 years and older).^12–15^ The previous literature suggests that various sociodemographic factors such as low socioeconomic status, household composition, disability, minority status, and housing all contribute to disaster vulnerability.^16,17^ In addition, a previous review established that females are more likely to have greater concerns and levels of fear about earthquakes than males.^17^ However, the findings do not suggest a specific cause of the health problems in the acute phase of disaster among women and the elderly. The previous review also stated that numerous uncertainties make it difficult to evaluate the effects of disaster on human health, including the paucity of high-quality epidemiological data and the unknown social, economic, and political responses.^18^

Regarding health event categories, the study results show that disaster stress-related symptoms, wounds, and skin illnesses accounted for more than 35% of overall consultations. In agreement with other studies, stress is a major health problem during the earthquakes, leading to increased weakness and vulnerability to not just physical sickness, but also poor mental health.^11^ Stress was the highest health event among patients aged 15-64 years and was the second highest health event among the elderly and children. This is in line with a previous disaster psychiatry study of four natural disasters that found patients aged 20-64 years accounted for a large proportion.^19^ Another study showed that older survivors were more likely to experience general psychiatric illness and posttraumatic stress disorder (PTSD) than the younger adults.^20^ Likewise, large numbers of children having significant symptoms of PTSD, peri-traumatic stress, depression, and anxiety have been reported.^21,22^ Children are especially poor at communicating their symptoms compared to adults and elderly people,^23^ and they may need more attention directly following a disaster. Turning to the time course, a substantial increase in the number of disaster stress-related symptoms on Day 1 and Day 2, which peaked on Day 3, was observed. This result is consistent with studies indicating a noticeable increase in mental health consultations from Day 0 to Day 2, which peaked over one week’s time.^24,25^ Although total health consultations decreased after Day14, disaster stress-related symptoms still accounted for a large proportion of the total (66.7%). This might be caused by the panic generated by an unexpected onset of events in the early days, as well as physical health issues, personnel losses, and economic difficulty in the following days. Therefore, EMTs should be aware not only of physical health issues, but also mental health problems and potential triggers among all generations immediately after a disaster, so as not to leave behind those who need mental health care.

Wounds are one of the most common health events recorded in a disaster response. In the case of earthquakes, 30%-50% of wounds are caused by falling and moving furniture.^26^ The current analysis revealed that wounds were more prevalent in patients aged 15-64 years or over 65 years. They can occur while people return to clean damaged homes, suffering skin injuries from treading on nails, getting fingers cut, or falling.^27^ A previous study found the risk of injury was 2.9-times higher for people over 65 years old.^12^ A diminished capacity to recover from harm or adopt preventative measures may be one factor. The number of wounds peaking at Day 2, after which the percentage of wound consultations statistically decreased, was observed. This is in line with a previous study in hospital settings indicating the highest consultation demand is within the first 24 hours;^28^ patients who are injured typically go to emergency rooms only for the first three to five days, after which hospital case patterns practically return to normal.^29^ Therefore, this high demand for trauma care immediately after the earthquake emphasizes the importance of establishing partnerships with EMTCC, including hospital transport, from the acute phase.

The study’s investigation found that skin disease was the third most prevalent health event among all consultations. Previous research revealed several skin diseases associated with earthquakes, such as traumatic, allergic, psychogenic, microbial, parasitic, and miscellaneous disorders.^30^ The J-SPEED records all types of skin problems as a “skin disease.” As a result, the analysis of the J-SPEED data doesn’t allow to be known exactly what kind of skin diseases occurred. The current result showed that skin disease was the highest health event among children aged 0-14 years, followed by disaster stress-related symptoms. A previous study on children with allergic dermatitis reported that symptoms of allergic disease such as eczema were associated with disaster-related stress due to changes in living environments after the earthquake.^31^ This context may help to explain the high proportion of skin disease and other disaster stress-related symptoms among children. Patients 15-64 years old were also vulnerable to skin diseases. One study suggests skin infections or traumatic skin disorder are linked to cleaning damaged homes.^27^ Another study revealed that elderly patients with limited mobility had a higher incidence of skin diseases such as pressure ulcers because of a scarcity of resources, manpower, and access to utilities.^32^ Skin diseases peaked twice on Day 5 and Day 11. No other reports of temporal transition of dermatological care during disasters were found. Future research and epidemiological study are needed on skin care needs during disasters, including temporal transition. By using J-SPEED and WHO EMT Minimum Data Set (MDS), which J-SPEED is based on and includes a skin disease item, for standardized medical data collection during and after a disaster, international comparisons are possible. Furthermore, the current findings suggest that the demand for dermatological care is high immediately after a disaster, and it has been reported that skin diseases in disasters have a significant impact on quality of life, even when the prognosis is not life-threatening.^33^ Taken together, increasing understanding of the triggers of skin problems and skin disease management for all generations is of great importance.

The key advantage of this research is the data gathering system, J-SPEED, a new standard data reporting system among EMTs in Japan. Real-time data reporting allows the EMTCC to conduct data-driven coordination throughout the response, as well as epidemiological studies that incorporate contextual data and improve the implementation of lessons gained.

## Limitations

In this study, there were some limitations. One weakness of the study was that J-SPEED reporting was initiated by EMTs rather than local health facilities, so not all clinical actions could be captured in the data. For example, only a few instances of dialysis (0.5% of total consultation) were recorded by EMTs in this study, despite dialysis being a primary problem related to continuous medical treatment in disaster response, particularly when a blackout occurs, as was the case across the entire Hokkaido region. Therefore, it should not be concluded that the medical needs of dialysis were low, because cases might have been brought directly to local hospitals. Relevant health hazards may be under-estimated as a result of these reporting factors. Second, J-SPEED reporting is still a new technique, and the reporting and data collecting process during in-field emergency response face a few obstacles, including a lack of pre-training and a lack of comprehension of reporting item definitions and J-SPEED reporting processes. Missing data and unregistered patients were common issues during data collecting with the J-SPEED. As a result, there was under-reporting, leading to an under-estimation of each health occurrence.

## Conclusions

A detailed assessment of 739 consultations is provided through a study of J-SPEED data from the Hokkaido Iburi Eastern Earthquake 2018, as reported by 32 EMTs. Disaster-related stress was the most often reported health event throughout the response period, followed by wounds and skin illnesses. The health effects of natural disasters are determined by a variety of factors, and the current study’s outcomes are extremely context specific; nevertheless, as more data are acquired in future research, it is expected that the consistency of findings will improve.
